# Deep learning based optic nerve sheath diameter characterization and structure quantification on transorbital ultrasound images

**DOI:** 10.3389/fmed.2025.1705459

**Published:** 2026-01-12

**Authors:** Miao Yang, Cong Liu, Pingyang Zou, Wu Wang

**Affiliations:** 1 Department of Anesthesiology, Guizhou Provincial People's Hospital, Guiyang, China; 2 School of Optical-Electrical and Computer Engineering, University of Shanghai for Science and Technology, Shanghai, China; 3 Electrical Engineering College, Guizhou University, Guiyang, China

**Keywords:** deep learning, optic nerve sheath diameter characterization, optic nerve segmentation, artificial intelligence, medical imaging

## Abstract

Optic nerve quantification plays a pivotal role as a biomarker in the non-invasive assessment of elevated intracranial pressure and other neuro-ophthalmic conditions. The manual identification of these optic nerve structures is both resource-intensive and time-consuming. The accuracy of optic nerve segmentation in automated methods directly depends on the quality of the ultrasound images. In instances of sub-optimal image quality, applying deep learning-based methodologies emerges as a more effective approach for precise segmentation. In this work, we propose a deep neural network combining the benefits of shared and specific feature extraction branches as well as the uncertainty-aware loss function. Such an uncertainty-aware loss function could enable the model to learn a robust object structure. Experiments on a multi-center publicly available dataset demonstrate the superior performance of our model in optic nerve segmentation and its strong potential of optic nerve sheath diameter quantification. Specifically, our model has achieved 73.3 % Dice score and 84.5% AUROC on the test dataset, outperforming the state-of-the-art models by a large margin.

## 1 Introduction

In recent years, the growing number of surgical patients, together with advancements in surgical techniques and the widespread adoption of minimally invasive procedures, has imposed higher demands on anesthesia and perioperative management. During general anesthesia, factors such as surgical manipulation, carbon dioxide pneumoperitoneum, and specific patient positioning can increase cerebral blood flow, elevate resistance to intracranial venous return, and raise jugular bulb pressure, thereby increasing intracranial pressure (ICP) (1–3). These changes may disrupt the balance between cerebral oxygen supply and demand, thereby increasing the risk of perioperative neurological complications. Cerebral protection is particularly critical in neurosurgery, in surgeries involving elderly patients, during emergency resuscitation, and cardiopulmonary-cerebral resuscitation following cardiac arrest.

Clinically, monitoring ICP in high-risk patients is essential. However, invasive ICP monitoring carries risks such as bleeding, infection, and catheter obstruction, in addition to being technically demanding and costly (4–7). The optic nerve sheath (ONS), an extension of the intracranial dura and arachnoid mater, contains a complex system of trabeculae and septa that compartmentalize the subarachnoid space, allowing cerebrospinal fluid (CSF) to flow gradually (8). Elevated ICP causes CSF to enter the ONS, leading to dilation of the optic nerve sheath diameter (ONSD), which serves as a reliable indicator of raised ICP (9–11). Studies have demonstrated a positive correlation between ultrasonographically measured ONSD and ICP (12–14), establishing it as a practical method for dynamic ICP monitoring (15) and even a potential alternative to CT in diagnosing intracranial hypertension (16). Transorbital ultrasound measurement of ONSD at 3 mm behind the retina (17) offers several advantages, including non-invasiveness, real-time assessment, rapidity, safety, high reproducibility, and bedside applicability (18, 19). This technique has broad clinical utility as an intraoperative ICP monitoring tool, providing clinicians with a novel approach to assess ICP dynamically. Early detection of elevated ICP can guide anesthesia management and perioperative decision-making, improving patient outcomes. Currently, there is no universally established ONSD threshold for intracranial hypertension due to variations across ethnicities and individuals (20, 21). Nevertheless, continuous ONSD monitoring can effectively track fluctuations in ICP. Multiple intraoperative factors, such as prolonged Trendelenburg positioning, carbon dioxide pneumoperitoneum (22–27), blood pressure, arterial carbon dioxide partial pressure, and anesthetic agents (28, 29), may influence ONSD. Anesthesiologists must closely monitor these variables to ensure accurate interpretation. By detecting ONSD changes earlier, proactive interventions can be implemented to mitigate ICP elevation, thereby preventing cerebral edema, ischemia, hypoxia, and perioperative neurological complications such as delayed emergence, postoperative delirium, and postoperative cognitive dysfunction.

Conventional ultrasonographic ONSD measurement requires operator expertise and is subject to inter-observer variability, making standardization a key concern in this field (30, 31). Inconsistent techniques may introduce measurement bias, compromising diagnostic reliability. Deep learning, a machine learning (ML) technique that extracts predictive features from labeled medical imaging datasets, circumvents complex manual processing. With the rapid advancement of artificial intelligence (AI), integrating AI into medical practice enables automated real-time assessments, significantly enhancing clinical workflows. AI-based algorithms (32) can automatically identify and measure ONSD in ultrasound images, precisely localizing its boundaries and calculating its diameter with high accuracy and consistency. Compared to manual measurements, AI reduces operator dependency and human error while improving efficiency (33). Real-time AI-assisted ONSD monitoring facilitates prompt detection of abnormal changes, enabling timely therapeutic adjustments and optimizing anesthesia management. Such an AI-assistant pipeline can reduce perioperative neurological complications, improve patient prognosis, and enhance overall perioperative safety. In this work, we propose a deep-learning-based method to automatically measure the ONSD via accurate optic nerve segmentation in real time. Our proposed method leverages the benefits of multi-scale attentions mechanism, combining the multitask-learning-based neural network framework with uncertainty-aware loss-based learning process. Significant improvement is observed over a multicenter dataset on both ONSD diameter quantification and optic nerve segmentation tasks over the compared state-of-the-art methods as well as the baseline models.

## 2 Related work

ML offers a promising solution to several challenges associated with manual ultrasonographic ONSD measurement, particularly by reducing reliance on expert operators. When trained on a sufficiently diverse dataset, ML algorithms may also help mitigate the variability in image acquisition and interpretation that is commonly reported in the literature (34).

For example, Pang et al. (35) were the first to apply deep learning, specifically convolutional neural networks (CNNs), to the task of automating ONSD measurement in ultrasound imaging. Their system operated by identifying the eyeball region, then estimating the optic nerve's direction using the centroids of the eye and optic nerve, before calculating ONSD based on segmentation of the hypoechoic zone located behind the eye. Differently, Meiburger et al. (33) use a single model to segment images from a multicenter data set with different US machines. This is the first time that ML algorithm shows the ability of fully automated pipeline in this task that can actually handle diverse imaging modalities and clinical settings. Similarly, Marzola et al. (36) trained a segmentation model that can generate relatively reliable results on segmentation task given region-based segmentation metrics, while the measurement of ONSD is lower than the expected, which may result in unstable diagnosis.

To improve, researchers proposed different methods to get better ONSD measures automatically. For example, Hirzallah et al. (37) trained CNN model to predict the mask between subarachnoid space's outer margins. This is the first time that a ML method can generate reliable performance on automatic ONSD measurement using mask instead of anatomic segmentation or landmark detection, while there is still limitation such as the inability to differentiate finer anatomic features. For example, it is very hard to differentiate the internal and external ONSD. Similarly, Netteland et al. (38) compared automated and manual ONSD measurements in patients with traumatic brain injury (TBI) admitted to a neurocritical care unit. Their method was the first to guide clinicians in correct probe placement during image acquisition. However, their method has suboptimal ON alignment on a subset of images, which may compromise accuracy. One potential solution is to incorporate real-time feedback mechanism for image quality assessment, while this may lead to additional subjective feedback or individual diversity. Differently, Singh et al. (39) created a region-based CNN object detection framework designed to automatically select the optimal frame for measurement by evaluating four factors: detection confidence, assigned class label, and specific criteria related to the window and sheath boundaries. Once the best frame was identified, the algorithm proceeded to locate the optic nerve along with its center and tip. From the ON tip, it generated multiple parabolic curves, ultimately selecting the parabola that exhibited the greatest variation in summed intensity to determine the ONSD measurement.

For ONSD measurement to be truly autonomous, both image acquisition and measurement must be automated. However, a major gap in the literature lies in the limited attention given to automating the acquisition process. Most existing approaches have concentrated on automating the measurement component, while still relying on skilled operators to capture high-quality ultrasound images. Addressing this limitation could involve using machine learning to provide real-time feedback and guidance to novice users during image acquisition (40), or leveraging robotic ultrasound systems to standardize the imaging process (40, 41). Future research should assess how well models perform when ONSD measurements are affected by common imaging artifacts–such as a tilted optic nerve axis, thickened outer bands, acoustic shadowing, and unclear anatomical boundaries. These artifacts can significantly hinder accurate image analysis. Therefore, developing models that either maintain measurement accuracy despite these challenges or can reliably flag suboptimal images as unsuitable for measurement would enhance their clinical utility and real-world applicability.

Differently, in this work, we propose a deep-learning-based automatic method to capture the anatomy structure as well as the quantitative result of ONSD at the same time. Using the proposed multi-scale attention pipeline and uncertainty-aware learning loss, our model can identify different scales of boundary area when low-quality issues occur, such as blurred boundaries. Our experimental results demonstrate the ability of our proposed method, regardless of qualitative or quantitative results. More details can be found in the Experiment section. Notably, our method is novel and different from the existing methods, which can share a similar pipeline with uncertainty estimation and attention mechanism. For example, compared with Meiburger et al. (33), our method can merge the uncertainty into the loss function calculation, where different potentials are considered. Differently, they can only directly estimate the final results while there is no estimation process of the uncertainty. On the other hand, compared with Marzola et al. (36), our model can fully utilize the attention mechanism to guide the model to focus on the region of the interested area, resulting in better segmentation performance.

## 3 Material and methods section

**Figure 1:**
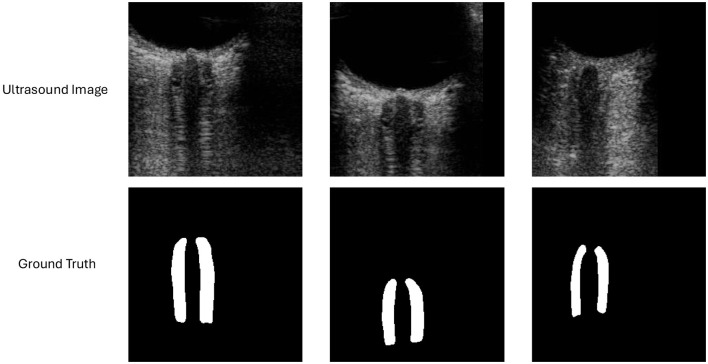
Sample ultrasound image for optic nerve segmentation and potential OND/ONSD quantification.

**Figure 2:**
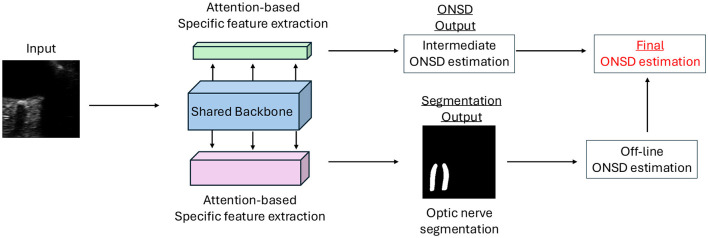
Figure of our proposed model structure. Our model contains three modules, including shared backbone model and attention-based specific feature extraction module for two tasks. The detailed structure of each module is shown in the figure. Notably, the final ONSD estimation is combined from both the direct ONSD estimation from the model and also the post-processed ONSD results after the segmentation performance of the model's output.

An example of ultrasound images and masks are shown in Figure 1. An overall model structure is introduced in Figure 2, where there is a shared feature extraction module, a specific feature extraction module, and an uncertainty-aware learning process. Specifically, the output from ON segmentation brunch will be fed into the ONSD direct estimation brunch, as prior knowledge, for reasonable estimation. The final estimation of ONSD will be the one that averaged from both ON brunch (after post-process) and direct ONSD estimation brunch.

### 3.1 Shared feature extraction module

Instead of relying on a conventional shared backbone to extract general-purpose features for multiple tasks, we adopted an attention-driven, task-adaptive backbone architecture that enhances the discriminative capacity of features for respective tasks, ultimately boosting the performance of the primary task. Notably, this is inspired by Meng et al. (42). The architecture is illustrated in Figure 2, comprising a central backbone and two attention-modulated branches, each tailored for specific tasks. For consistency and fair comparison with the existing methods, our backbone leverages the ResNet50 (43) architecture. However, this choice is flexible; alternative robust backbones have also been explored. To incorporate global contextual dependencies, we devised a Feature Aggregation Block, which hierarchically and iteratively integrates features from deeper layers back into earlier ones. This process involves up-sampling (via bilinear interpolation), concatenation, and convolution to align feature map channels and spatial resolutions. The aggregation process enables the network to capture a rich spectrum of semantic and spatial information spanning multiple levels of abstraction.

Within each of the two specific task branches, the multi-scale attention mechanism is employed to refine task-specific feature learning. Each attention block begins with global average pooling, which summarizes global context across channels, generating an attention tensor to modulate feature emphasis. This is followed by two blocks, each containing a 1 × 1 convolution layer, batch normalization (44), and non-linear activations (ReLU and sigmoid). The attention score is then applied via element-wise multiplication to modulate the output of the shared backbone, effectively filtering out irrelevant features for each task. This design enables the backbone to retain generalizable representations, while the branches learn task-specific features in a unified, end-to-end framework.

### 3.2 Specific feature extraction module

The attention mechanism in our model is crafted to help the task-specific branches focus on features relevant to their particular tasks. This is achieved by introducing a soft attention mask that modulates the shared features in the network. For each task and each feature channel, a distinct attention mask is applied. Let's denote the shared feature maps at the ℓ^th^ layer as **Q**^(ℓ)^, and the corresponding attention mask for task *t* at that layer as ${\text{M}}_{t}^{\left(\ell \right)}$. The task-specific feature maps, denoted as ${\text{F}}_{t}^{\left(\ell \right)}$, are then obtained by performing an element-wise multiplication:


$$\begin{array}{l} {\text{F}}_{t}^{\left(\ell \right)}={\text{M}}_{t}^{\left(\ell \right)}⊙{\text{Q}}^{\left(\ell \right)}, \end{array}$$ (1)

where ⊙ denotes element-wise multiplication.

In the initial layers, the attention mechanism receives as input only the shared features extracted by the base network. However, for subsequent layers (ℓ≥2), the attention modules incorporate both the shared features **U**^(ℓ)^ and the task-specific features from the preceding layer, ${\text{F}}_{t}^{\left(\ell -1\right)}$, using a combination of convolutional transformations and concatenations:


$$\begin{array}{l} {\text{M}}_{t}^{\left(\ell \right)}=H_{t}^{\left(\ell \right)}\left(G_{t}^{\left(\ell \right)}\left(\left[{\text{U}}^{\left(\ell \right)};F^{\left(\ell \right)}\left({\text{F}}_{t}^{\left(\ell -1\right)}\right)\right]\right)\right). \end{array}$$ (2)

Here, *F*^(ℓ)^, $G_{t}^{\left(\ell \right)}$, and $H_{t}^{\left(\ell \right)}$ represent convolutional blocks equipped with batch normalization and non-linear activation functions. The functions $G_{t}^{\left(\ell \right)}$ and $H_{t}^{\left(\ell \right)}$ use 1 × 1 convolution kernels to compute the attention masks specific to each task, while *F*^(ℓ)^ employs 3 × 3 kernels to refine and aggregate features across channels and spatial dimensions. Downsampling or pooling is applied to align the resolutions of the feature maps where needed.

To ensure that the attention mask outputs are valid for modulating features, we apply a sigmoid activation to ${\text{M}}_{t}^{\left(\ell \right)}$, constraining its values between 0 and 1. This design ensures that, at worst, our system performs comparably to a standard shared multi-task model where tasks are split only at the final stage.

### 3.3 Uncertainty-aware loss functions

Region-based loss functions, such as the Dice loss (45), are widely employed due to their effectiveness in achieving accurate segmentation outcomes. Nevertheless, their performance can be compromised by the complex and noisy background typically present in ultrasound images. This challenge often leads to uncertainty near the boundaries of the region of interest, as noted in et al. (46), potentially resulting in suboptimal segmentation quality and less reliable estimation of the optic nerve diameter sheath characterization. In response, we incorporated the Uncertainty-aware auto learning loss (47, 48), which allows the model to adjust its learning process based on detected uncertainties, improving both resilience and precision by emphasizing confident predictions and reducing the impact of ambiguous ones. By incorporating uncertainty into the loss calculation, the model can dynamically assign weights to different components of the loss function, helping to balance global and local accuracy while mitigating the effects of class imbalance. As a result, the model focuses more on reliable data points and minimizes the influence of potentially noisy or uncertain samples, thereby enhancing robustness. Ultimately, this leads to a noticeable improvement in segmentation performance, as the model becomes better equipped to handle both confident and uncertain predictions effectively. The uncertainty-aware loss *L*_*uncertainty*_ is formulated as following:


$$\begin{array}{l} L_{uncertainty}=\overset{K}{\underset{k=1}{\sum }}\left(\sigma_{k}\times L_{k}+\log\left(1+\sigma_{k}\right)\right). \end{array}$$ (3)

Here, *K* denotes the total number of individual loss components, where each *L*_*k*_ represents a specific loss term, such as Dice loss (45), cross entropy loss, mean squared error, or shape-distance loss. The σ_*k*_ terms are learnable noise parameters via a MLP at the output layer of the model. A higher noise level indicates greater uncertainty in understanding the data, which in turn assigns a higher learning weight to that component. These parameters are optimized during training to minimize the overall loss. The final term in the loss function acts as a regularization mechanism, preventing the noise levels from becoming excessively large. In this work, we incorporate several key loss functions, while additional options can be found in the survey (49):

- Mean Squared Error Loss: A pixel-wise loss function that calculates the squared difference between each predicted pixel value and its corresponding ground truth. It ensures pixel-level accuracy and quantifies the model's prediction precision.
- Dice Coefficient Loss: A region-based loss function focusing on the overlap between the predicted segmentation and the ground truth, promoting accurate delineation of shapes and boundaries in the segmented regions.
- Cross-Entropy Loss: A distribution-based loss that measures the classification accuracy at the pixel level, helping improve the model's ability to distinguish between different classes.
- Shape-Distance Loss: A distribution-based loss designed to enhance shape features and improve boundary alignment in segmentation tasks. It encourages the model to focus on the geometric and structural details of the target regions during training.

Notably, the above loss function term will only be used for the segmentation task of optic nerve, and we adopt different losses options (*L*_*regression*_) for the regression task of ONSD estimation brunch. Thus, the total loss function (*L*_*total*_) can be written as:


$$\begin{array}{l} L_{total}=w_{1}\times L_{regression}+W_{2}\times L_{uncertainty}, \end{array}$$ (4)

where *w*_1_ and *w*_2_ are the hyper-parameters that control the balanced weights of two loss terms, and we set them to 0.5 each in this work. Also, we adopt mean square error loss for the *L*_*regression*_ loss in this work after empirically validated.

### 3.4 Experiments

#### 3.4.1 Datasets

For this study, we employed the transorbital sonography (TOS) dataset (36). The dataset consists of 464 B-mode ultrasound images obtained from 110 individual participants. Data collection was performed across four independent medical centers using five different ultrasound machines, introducing a diverse range of imaging conditions and device-specific characteristics. To standardize the input for model development, all images were uniformly resized to 256 × 256 pixels and pre-cropped to focus on the orbital region. Expert manual annotations are provided for each image, delineating both the optic nerve (ON) and the optic nerve sheath (ONS). The multicenter, multi-device nature of the dataset enhances its utility in developing generalizable deep learning models for optic nerve assessment in transorbital ultrasound imaging.

#### 3.4.2 Experimental setting

To enhance the model's generalization ability and mitigate the risk of overfitting, we adopted an online data augmentation approach during training. This involved applying random geometric transformations—including horizontal flips and rotations—each with a 30% probability. The rotation angles were uniformly drawn from the range of −20° to 20°, allowing the model to learn orientation-invariant features and improving its robustness to anatomical variability. We did extensive experiments to tune the various hyperparameters, such as the augmentation policy including cropping, brightness scaling, etc. It turns out that different augmentation strategies did not make a very big difference to the final results. Model optimization was carried out using stochastic gradient descent (SGD) with a momentum term set to 0.9, which served to accelerate convergence and smooth the optimization trajectory. All experimental configurations were trained for 150 epochs. The initial learning rate was set at 5 × 10^−3^ and was progressively reduced using a step-decay policy, with a decay factor of 0.99 applied every 100 iterations to promote stable convergence. A batch size of 24 was used, providing a good trade-off between memory efficiency and gradient estimation stability. All experiments were executed in an end-to-end training manner on a high-performance workstation powered by an NVIDIA GeForce RTX 2080Ti GPU with 11GB of VRAM. For segmentation output, a fixed threshold of 0.5 was applied to the predicted probability maps to obtain binary masks. To ensure fair benchmarking and reproducibility, no post-processing steps or model ensembling strategies were employed throughout the evaluation pipeline. Notably, we performed five-fold experiments to demonstrate the fair and robust model's performance.

#### 3.4.3 Evaluation metrics

The model generates a probability map as its output for segmentation task, assigning each pixel a likelihood of belonging to the vessel class. In our experiments, a threshold of 0.5 was applied to the probability map to generate the final segmentation results. To thoroughly evaluate the performance of the proposed framework during the testing phase, we will compute the following metrics:

- SE (sensitivity) = TP / (TP + FN),
- SP (specificity) = TN / (TN + FP)
- F1 (F1 score) = (2 × TP) / (2 × TP + FP + FN)
- 95% HD = 95 % Hausdorff Distance
- AUROC = Area under the Receiver Operating Characteristic Curve.

In this context, correctly identifying a vessel pixel is termed a true positive (TP), while incorrectly classifying a non-vessel pixel as a vessel is referred to as a false positive (FP). Similarly, accurately recognizing a non-vessel pixel is called a true negative (TN), and failing to detect a vessel pixel is classified as a false negative (FN). The Hausdorff distance measures the greatest distance from a point in one set to the closest point in the other set. It captures the worst-case scenario of mismatch between predicted and ground truth boundaries. And 95% HD considers the distance that 95% of the points are within, effectively discarding the top 5% of outliers. This makes it more robust to extreme outliers, which can occur due to small segmentation errors.

#### 3.4.4 Compared methods

We compared our approach to other classic and state-of-the-art models that have achieved promising performance on different medical image segmentation tasks. All of the experiments are conducted under the same experimental setting. The compared methods are briefly introduced below:

- Unet (50): Unet is a convolutional neural network (CNN) architecture built for image segmentation tasks. Its distinctive U-shaped structure consists of an encoder that captures features and a corresponding decoder that reconstructs segmented outputs. Skip connections link matching layers in the encoder and decoder, preserving detailed information and context. This design has proven highly effective, particularly in biomedical image segmentation tasks.
- Unet++ (51): Unet++ is an enhanced variant of the U-Net model, designed for image segmentation tasks. It introduces nested skip connections and sophisticated feature aggregation pathways, enabling improved integration of multi-scale information and richer contextual understanding. These improvements contribute to more accurate and precise segmentation compared to the original U-Net design.
- Swin-Transformer (52): Swin-Transformer is a hierarchical vision transformer architecture that processes image patches at multiple scales using shifted windows. This approach enables the model to capture both local and global contextual information more effectively. With its innovative design, Swin-Transformer has achieved strong segmentation results while maintaining computational efficiency.
- AttenUnet (53): AttenUnet builds upon the classic U-Net model by introducing attention mechanisms that allow the network to concentrate on critical regions within an image during segmentation. These mechanisms help the model refine the outlines of objects and reduce distractions from less relevant details. This approach is especially valuable in tasks like medical image segmentation, where precise boundary definition is crucial.
- TransUnet (54): TransUNet is designed to enhance medical image segmentation by addressing the challenges faced by the commonly used U-Net architecture. It combines the ability of Transformers to capture global context with U-Net's strength in accurately locating features. The transformer component processes patches extracted from CNN feature maps to understand the broader context, and the decoder then integrates this information with high-resolution feature maps, resulting in more precise segmentation.
- Swin-Unet (55): Swin-Unet is a Unet-like pure transformer for medical image segmentation. The tokenised image patches are fed into the transformer-based U-shaped encoder-decoder architecture with skip-connections for local-global semantic feature learning. Their method can benefit from both the strength of Unet and Swin-Transformer for sufficient feature extraction and local-global context information aggregation.

## 4 Results

### 4.1 Optic nerve segmentation performance

**Figure 3:**
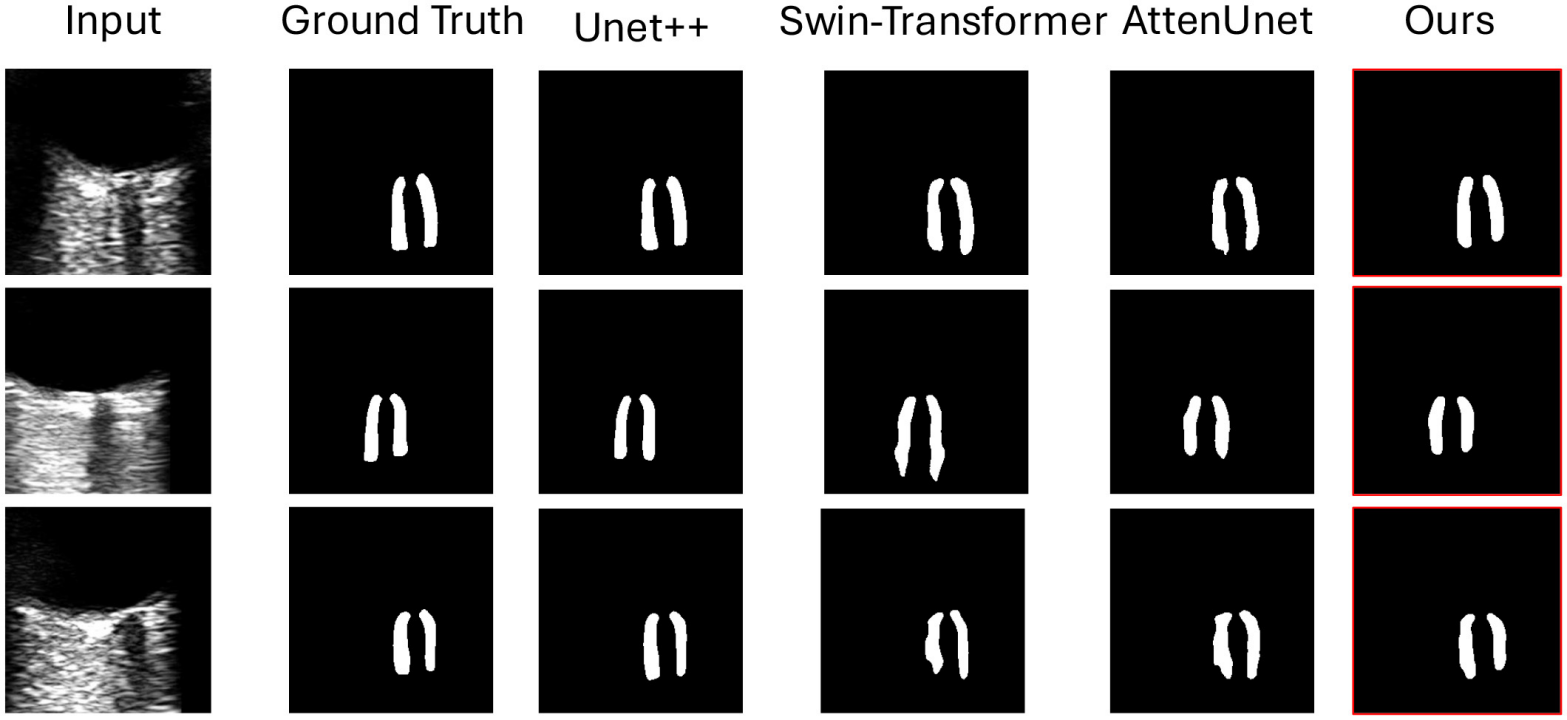
Qualitative results of the optic nerve segmentation. We compare our model with Unet++ (51), Swin-Transformer (52), AttenUnet (53). Our method can produce more accurate segmentation results than the other methods compared with the ground truth.

Figure 3 illustrates qualitative comparison with other compared methods on the test dataset. Table 1 shows the quantitative performance of *Ours* and other methods on four different datasets, respectively.

**Table 1:** Quantitative results comparison between our methods and other compared state-of-the-art methods on optic nerve segmentation.

**Methods**	**(95%) HD**	**SE**	**SP**	**Dice**	**AUROC**
*Unet*	26.1	92.2	96.5	72.2	83.2
*Unet++*	25.2	92.1	95.3	71.2	82.1
*Swin-Transformer*	23.3	91.0	95.3	71.0	80.0
*AttenUnet*	23.9	90.3	95.1	69.8	81.3
*TransUnet*	24.1	90.8	95.4	70.4	82.5
*Ours*	**21.2**	**94.1**	**96.9**	**73.3**	**84.5**

Performance is reported with (95%) *HD, SE, SP, Dice* and *AUROC*. The best performance is highlighted in bold.

In detail, *Ours* achieved 21.2 *(95%)HD* on optic nerve segmentation, which outperformed *Unet* (50) by 18.8 %, outperformed *Swin-Transformer* (52) by 9 % and outperformed *TransUnet* (54) by 12 %. *Ours* achieved 84.5 % *AUROC* on optic nerve segmentation, which outperformed *Unet* (50) by 1.6 %, outperformed *Swin-Transformer* (52) by 5.6 % and outperformed *TransUnet* (54) by 2.4 %. Additionally, *Ours* achieved 73.3 % *Dice* on optic nerve segmentation, which outperformed *Unet* (50) by 1.5 %, outperformed *Swin-Transformer* (52) by 3.2 % and outperformed *TransUnet* (54) by 4.1 %.

Notably, *Ours* adopted ResNet50 (43) as the backbone to extract the features from the input image, while some of the compared methods use Transformer-based architecture. Even though Transformer-based methods usually can obtain a better performance due to their superior ability of extracting feature, *Ours* benefits from the special designed module structure, thus can give a better performance.

Figure 3 shows the qualitative comparison between ours and other compared methods. It demonstrated that our proposed methods can segment the optic nerve boundary more accurately. This is important for optic nerve segmentation tasks and ONSD quantification with more accurate diameter calculation.

### 4.2 OND and ONSD estimation performance

To determine whether the enhancements in segmentation quality contribute to improved clinical measurement accuracy, we assessed both the optic nerve diameter (OND) and the optic nerve sheath diameter (ONSD). These measurements were derived using the standardized MATLAB tool accompanying the dataset. Consistent with previous methodologies (36), we extracted the diameters automatically from the predicted segmentation masks and benchmarked them against manually annotated ground truths.

Table 2 summarizes the quantitative findings. *Ours* achieved a mean absolute error (MAE) of 0.26 mm for OND and 0.41 mm for ONSD. Compared to Marzola et al. (36), our method shows a significant improvement in OND accuracy while maintaining comparable performance for ONSD. Our method's performance on intraclass correlation coefficients (ICC) were robust for both estimation measurements (OND: 0.67, ONSD: 0.66), indicating strong consistency aligning with clinical expert assessments.

**Table 2:** ONSD and OND measurement results comparison.

**Methods**	**MAE**		**ICC**		**Pearson**		**Error**	
	**OND**	**ONSD**	**OND**	**ONSD**	**OND**	**ONSD**	**OND**	**ONSD**
*Unet*	0.30	0.42	0.62	0.63	0.67	0.65	0.10	0.022
*Unet++*	0.34	0.41	0.63	0.64	0.66	0.65	0.11	0.021
*Swin-Transformer*	0.29	0.43	0.62	0.63	0.66	0.64	0.11	0.022
*AttenUnet*	0.31	0.42	0.65	0.64	0.65	0.64	0.12	0.023
*TransUnet*	0.32	0.40	0.63	0.65	0.66	0.65	0.12	0.022
*Ours*	**0.26**	**0.41**	**0.67**	**0.66**	**0.68**	**0.68**	**0.097**	**0.019**

MAE, mean absolute error (mm); ICC, intraclass correlation coefficient; Pearson, Pearson correlation against manual annotations; OND, optic nerve diameter; ONSD, optic nerve sheath diameter.

Both OND and ONSD are considered clinically relevant biomarkers, especially for detecting raised intracranial pressure (56). Our results confirm that the proposed uncertainty-aware architecture enhances segmentation quality and supports accurate, automated quantification of both anatomical structures from transorbital ultrasound images.

## 5 Discussion

Even though our model achieved superior performance over other methods in the test dataset, there are some limitations about the proposed methods and experimental design in the work. Firstly, the limited number of data makes the whole framework less generalizable and lacks a demonstration of robustness. Secondly, only one center of the dataset is used for this study, while in the real world, different scenes result in different image quality and conditions. Thus it is unknown if the model can handle the domain difference issue. Thirdly, the efficiency and inference speed are not addressed in this work, which could be important for a specific surgical task.

As for the future work, we will conduct validation experiments on more centers' datasets and more number of data cases included for more robust model's development.

## 6 Conclusion

We have proposed a novel and comprehensive framework for optic nerve segmentation and optic nerve sheath diameter quantification. It takes advantage of task-specific attention branches to aggregate the information while maintaining the accuracy of the boundary segmentation with the benefits of uncertainty-aware loss functions. Our experiments on multi-center large-scale dataset have demonstrated that our framework can simultaneously conduct accurate segmentation and potential optic nerve sheath diameter quantification performance.

## Data Availability

The original contributions presented in the study are included in the article/supplementary material, further inquiries can be directed to the corresponding author/s.

## References

[1] Halverson A Barrett W Iglesias A Lee W Garber S Sackier J. Decreased cerebrospinal fluid absorption during abdominal insufflation. Surg Endosc. (1999) 13:797–800. doi: 10.1007/s004649901102 10430688

[2] Lovell AT Marshall AC Elwell CE Smith M Goldstone JC. Changes in cerebral blood volume with changes in position in awake and anesthetized subjects. Anesth Analg. (2000) 90:372–6. doi: 10.1213/00000539-200002000-00025 10648324

[3] Petersen LG Petersen JCG Andresen M Secher NH Juhler M. Postural influence on intracranial and cerebral perfusion pressure in ambulatory neurosurgical patients. Am J Physiol-Regul Integr Comp Physiol. (2016) 310:R100–4. doi: 10.1152/ajpregu.00302.2015 26468260

[4] Tavakoli S Peitz G Ares W Hafeez S Grandhi R. Complications of invasive intracranial pressure monitoring devices in neurocritical care. Neurosurg Focus. (2017) 43:E6. doi: 10.3171/2017.8.FOCUS17450 29088962

[5] Chatzi M Karvouniaris M Makris D Tsimitrea E Gatos C Tasiou A . Bundle of measures for external cerebral ventricular drainage-associated ventriculitis. Crit Care Med. (2014) 42:66–73. doi: 10.1097/CCM.0b013e31829a70a5 23982025

[6] Chesnut RM Temkin N Carney N Dikmen S Rondina C Videtta W . A trial of intracranial-pressure monitoring in traumatic brain injury. N Engl J Med. (2012) 367:2471–81. doi: 10.1056/NEJMoa1207363 23234472 PMC3565432

[7] Le Roux P Menon DK Citerio G Vespa P Bader MK Brophy GM . Consensus summary statement of the international multidisciplinary consensus conference on multimodality monitoring in neurocritical care: a statement for healthcare professionals from the Neurocritical Care Society and the European Society of Intensive Care Medicine. Neurocrit Care. (2014) 21:1–26. doi: 10.1007/s12028-014-0041-5 25208678 PMC10596301

[8] Killer H Laeng H Flammer J Groscurth P. Architecture of arachnoid trabeculae, pillars, and septa in the subarachnoid space of the human optic nerve: anatomy and clinical considerations. Br J Ophthalmol. (2003) 87:777–81. doi: 10.1136/bjo.87.6.777 12770980 PMC1771732

[9] Dubourg J Javouhey E Geeraerts T Messerer M Kassai B. Ultrasonography of optic nerve sheath diameter for detection of raised intracranial pressure: a systematic review and meta-analysis. Intensive Care Med. (2011) 37:1059–68. doi: 10.1007/s00134-011-2224-2 21505900

[10] Dong J Li Q Wang X Fan Y A. review of the methods of non-invasive assessment of intracranial pressure through ocular measurement. Bioengineering. (2022) 9:304. doi: 10.3390/bioengineering9070304 35877355 PMC9312000

[11] Fernando SM Tran A Cheng W Rochwerg B Taljaard M Kyeremanteng K . Diagnosis of elevated intracranial pressure in critically ill adults: systematic review and meta-analysis. BMJ. (2019) 366:l4225. doi: 10.1136/bmj.l4225 31340932 PMC6651068

[12] Robba C Donnelly J Cardim D Tajsic T Cabeleira M Citerio G . Optic nerve sheath diameter ultrasonography at admission as a predictor of intracranial hypertension in traumatic brain injured patients: a prospective observational study. J Neurosurg. (2019) 132:1279–85. doi: 10.3171/2018.11.JNS182077 30849751

[13] Robba C Cardim D Tajsic T Pietersen J Bulman M Donnelly J . Ultrasound non-invasive measurement of intracranial pressure in neurointensive care: a prospective observational study. PLoS Med. (2017) 14:e1002356. doi: 10.1371/journal.pmed.1002356 28742869 PMC5526499

[14] Herpertz GU Focken P Radke O. Learning and reproducibility of ultrasonographic assessment of the optic nerve sheath diameter: a cohort study. Eur J Anaesthesiol Intens Care. (2025) 4:e0074. doi: 10.1097/EA9.0000000000000074 40453485 PMC12122177

[15] Wang Lj Chen Lm Chen Y Bao Ly Zheng Nn Wang Yz . Ultrasonography assessments of optic nerve sheath diameter as a noninvasive and dynamic method of detecting changes in intracranial pressure. JAMA Ophthalmol. (2018) 136:250–6. doi: 10.1001/jamaophthalmol.2017.6560 29392301 PMC5885896

[16] Kaur A Gautam PL Sharma S Singh VP Sharma S. Bedside ultrasonographic assessment of optic nerve sheath diameter as a means of detecting raised intracranial pressure in neuro-trauma patients: a cross-sectional study. Ann Indian Acad Neurol. (2021) 24:63–8. doi: 10.4103/aian.AIAN_51_20 33911381 PMC8061509

[17] Hirzallah MI Lochner P Hafeez MU Lee AG Krogias C Dongarwar D . Optic nerve sheath diameter point-of-care Ultrasonography Quality Criteria Checklist: an International Consensus Statement on Optic nerve sheath diameter imaging and measurement. Crit Care Med. (2024) 52:1543–56. doi: 10.1097/CCM.0000000000006345 38836697

[18] Koziarz A Sne N Kegel F Alhazzani W Nath S Badhiwala JH . Optic nerve sheath diameter sonography for the diagnosis of increased intracranial pressure: a systematic review and meta-analysis protocol. BMJ Open. (2017) 7:e016194. doi: 10.1136/bmjopen-2017-016194 28801417 PMC5629711

[19] Pansell J Bell M Rudberg P Friman O Cooray C. Optic nerve sheath diameter measurement by ultrasound: evaluation of a standardized protocol. J Neuroimaging. (2022) 32:104–10. doi: 10.1111/jon.12936 34555223

[20] Chen H Ding GS Zhao YC Yu RG Zhou JX. Ultrasound measurement of optic nerve diameter and optic nerve sheath diameter in healthy Chinese adults. BMC Neurol. (2015) 15:1–6. doi: 10.1186/s12883-015-0361-x 26148482 PMC4493801

[21] Bäuerle J Lochner P Kaps M Nedelmann M. Intra-and interobsever reliability of sonographic assessment of the optic nerve sheath diameter in healthy adults. J Neuroimaging. (2012) 22:42–5. doi: 10.1111/j.1552-6569.2010.00546.x 21121998

[22] Robba C Cardim D Donnelly J Bertuccio A Bacigaluppi S Bragazzi N . Effects of pneumoperitoneum and Trendelenburg position on intracranial pressure assessed using different non-invasive methods. BJA: Br J Anaesth. (2016) 117:783–91. doi: 10.1093/bja/aew356 27956677

[23] Ustick JJ Pardon LP Chettry P Patel NB Cheng H. Effects of head-down tilt on optic nerve sheath diameter in healthy subjects. Ophthal Physiol Opt. (2023) 43:1531–9. doi: 10.1111/opo.13200 37401194 PMC10592427

[24] Chin JH Seo H Lee EH Lee J Hong JH Hwang JH . Sonographic optic nerve sheath diameter as a surrogate measure for intracranial pressure in anesthetized patients in the Trendelenburg position. BMC Anesthesiol. (2015) 15:1–6. doi: 10.1186/s12871-015-0025-9 25861241 PMC4389861

[25] Dip F Nguyen D Rosales A Sasson M Menzo EL Szomstein S . Impact of controlled intraabdominal pressure on the optic nerve sheath diameter during laparoscopic procedures. Surg Endosc. (2016) 30:44–9. doi: 10.1007/s00464-015-4159-0 25899811

[26] Yashwashi T Kaman L Kajal K Dahiya D Gupta A Meena SC . Effects of low-and high-pressure carbon dioxide pneumoperitoneum on intracranial pressure during laparoscopic cholecystectomy. Surg Endosc. (2020) 34:4369–73. doi: 10.1007/s00464-019-07207-w 31617096

[27] Hasirci I Ulutas E Polat A Harb A Tire Y Kartal A. Comparison of extraperitoneal and intraperitoneal laparoscopic procedures for intracranial pressure increase: a prospective clinical study. Eur Rev Med Pharmacol Sci. (2023) 27:6207–14. doi: 10.26355/eurrev_202307_32979 37458626

[28] Zhu T Yuan C Qian M Zhao L Li H Xie Y. Effect of dexmedetomidine on intracranial pressure in patients undergoing gynecological laparoscopic surgery in Trendelenburg position through ultrasonographic measurement of optic nerve sheath diameter. Am J Transl Res. (2022) 14:6349. 36247291 PMC9556444

[29] Kim JE Koh SY Jun IJ. Comparison of the effects of propofol and sevoflurane anesthesia on optic nerve sheath diameter in robot-assisted laparoscopic gynecology surgery: a randomized controlled trial. J Clin Med. (2022) 11:2161. doi: 10.3390/jcm11082161 35456254 PMC9024447

[30] Montorfano L Yu Q Bordes SJ Sivanushanthan S Rosenthal RJ Montorfano M. Mean value of B-mode optic nerve sheath diameter as an indicator of increased intracranial pressure: a systematic review and meta-analysis. Ultrasound J. (2021) 13:1–12. doi: 10.1186/s13089-021-00235-5 34215966 PMC8253877

[31] Hirzallah MI Lochner P Hafeez MU Lee AG Krogias C Dongarwar D . Quality assessment of optic nerve sheath diameter ultrasonography: scoping literature review and Delphi protocol. J Neuroimaging. (2022) 32:808–24. doi: 10.1111/jon.13018 35711135

[32] Chu Y Xu J Wu C Ye J Zhang J Shen L . Optic nerve sheath ultrasound image segmentation based on CBC-YOLOv5s. Electronics. (2024) 13:3595. doi: 10.3390/electronics13183595

[33] Meiburger KM Naldi A Lochner P Marzola F. Automatic segmentation of the optic nerve in transorbital ultrasound images using a deep learning approach. In: 2021 IEEE International Ultrasonics Symposium (IUS). Xi'an: IEEE (2021). p. 1-4. doi: 10.1109/IUS52206.2021.9593827

[34] Escamilla-Oca nas CE Morales-Cardona NC Sagreiya H Akhbardeh A Hirzallah MI. Automation of ultrasonographic optic nerve sheath diameter measurement: a scoping review. J Neuroimaging. (2025) 35:e70017. doi: 10.1111/jon.70017 39853865

[35] Pang M Liu S Lin F Liu S Tian B Yang W . Measurement of optic nerve sheath on ocular ultrasound image based on segmentation by CNN. In: 2019 IEEE International Conference on Signal, Information and Data Processing (ICSIDP). Chongqing: IEEE (2019). p. 1–5. doi: 10.1109/ICSIDP47821.2019.9173198

[36] Marzola F Lochner P Naldi A Lemor R Stögbauer J Meiburger KM. Development of a deep learning-based system for optic nerve characterization in transorbital ultrasound images on a multicenter data set. Ultrasound Med Biol. (2023) 49:2060–71. doi: 10.1016/j.ultrasmedbio.2023.05.011 37357081

[37] Hirzallah MI Bose S Hu J Maltz JS. Automation of ultrasonographic optic nerve sheath diameter measurement using convolutional neural networks. J Neuroimaging. (2023) 33:898–903. doi: 10.1111/jon.13163 37845814

[38] Netteland DF Aarhus M Smistad E Sandset EC Padayachy L Helseth E . Noninvasive intracranial pressure assessment by optic nerve sheath diameter: automated measurements as an alternative to clinician-performed measurements. Front Neurol. (2023) 14:1064492. doi: 10.3389/fneur.2023.1064492 36816558 PMC9928958

[39] Singh M Kumar B Agrawal D. Good view frames from ultrasonography (USG) video containing ONS diameter using state-of-the-art deep learning architectures. Med Biol Eng Comput. (2022) 60:3397–417. doi: 10.1007/s11517-022-02680-3 36190609

[40] Narang A Bae R Hong H Thomas Y Surette S Cadieu C . Utility of a deep-learning algorithm to guide novices to acquire echocardiograms for limited diagnostic use. JAMA Cardiol. (2021) 6:624–32. doi: 10.1001/jamacardio.2021.0185 33599681 PMC8204203

[41] Jiang Z Salcudean SE Navab N. Robotic ultrasound imaging: state-of-the-art and future perspectives. Med Image Anal. (2023) 89:102878. doi: 10.1016/j.media.2023.102878 37541100

[42] Meng Y Bridge J Zhao Y Joddrell M Qiao Y Yang X . Transportation object counting with graph-based adaptive auxiliary learning. IEEE Trans Intell Transport Syst. (2022) 24:3422–37. doi: 10.1109/TITS.2022.3226504

[43] He K Zhang X Ren S Sun J. Deep residual learning for image recognition. In: Proceedings of the IEEE Conference on Computer Vision and Pattern Recognition. Las Vegas, NV (2016). p. 770–8. doi: 10.1109/CVPR.2016.90

[44] Ioffe S Szegedy C. Batch normalization: accelerating deep network training by reducing internal covariate shift. In: Proceedings of the 32nd International Conference on Machine Learning. Lille (2015). p. 448–56.

[45] Drozdzal M Vorontsov E Chartrand G Kadoury S Pal C. The importance of skip connections in biomedical image segmentation. In: Deep Learning and Data Labeling for Medical Applications. Springer (2016). p. 179–87. doi: 10.1007/978-3-319-46976-8_19

[46] Meng Y Zhang Y Xie J Duan J Joddrell M Madhusudhan S . Multi-granularity learning of explicit geometric constraint and contrast for label-efficient medical image segmentation and differentiable clinical function assessment. Med Image Analy. (2024) 95:103183. doi: 10.1016/j.media.2024.103183 38692098

[47] Hu S Wang X Lyu S. Rank-based decomposable losses in machine learning: a survey. IEEE Trans Pattern Anal Mach Intell. (2023) 45:13599–620. doi: 10.1109/TPAMI.2023.3296062 37459267

[48] Wang X Liu X Huang P Huang P Hu S Zhu H. U-medsam: uncertainty-aware medsam for medical image segmentation. In: Medical Image Segmentation Challenge. Seattle, WA: Springer (2024). p. 206–17. doi: 10.1007/978-3-031-81854-7_14

[49] Jadon S. A survey of loss functions for semantic segmentation. In: 2020 IEEE Conference on Computational Intelligence in Bioinformatics and Computational Biology (CIBCB). Viña del Mar: IEEE (2020). p. 1–7. doi: 10.1109/CIBCB48159.2020.9277638

[50] Ronneberger O Fischer P Brox T. U-net: convolutional networks for biomedical image segmentation. In: International Conference on Medical Image Computing and Computer-Assisted Intervention. Munich: Springer (2015). p. 234–41. doi: 10.1007/978-3-319-24574-4_28

[51] Zhou Z Siddiquee MMR Tajbakhsh N Liang J. UNet++: a nested U-net architecture for medical image segmentation. In: Deep Learning in Medical Image Analysis and Multimodal Learning for Clinical Decision Support. Grananda: Springer (2018). p. 3–11. doi: 10.1007/978-3-030-00889-5_1 PMC7329239 32613207

[52] Liu Z Lin Y Cao Y Hu H Wei Y Zhang Z . Swin transformer: hierarchical vision transformer using shifted windows. In: Proceedings of the IEEE/cvf International Conference on Computer Vision. Montreal, QC: IEEE (2021). p. 10012–22. doi: 10.1109/ICCV48922.2021.00986

[53] Oktay O Schlemper J Le Folgoc L Lee M Heinrich M Misawa K . Attention U-net: learning where to look for the pancreas. In: Medical Imaging with Deep Learning. Zurich (2022).

[54] Chen J., Mei, J., Li, X., Lu, Y., Yu, Q., Wei, Q., et al. (2024). TransUNet: rethinking the U-Net architecture design for medical image segmentation through the lens of transformers. Med. Image Anal. 97:103280. doi: 10.1016/j.media.2024.103280 39096845

[55] Cao H Wang Y Chen J Jiang D Zhang X Tian Q . Swin-unet: Unet-like pure transformer for medical image segmentation. In: European Conference on Computer Vision. Tel Aviv: Springer (2022). p. 205–18. doi: 10.1007/978-3-031-25066-8_9

[56] Lochner P Czosnyka M Naldi A Lyros E Pelosi P Mathur S . Optic nerve sheath diameter: present and future perspectives for neurologists and critical care physicians. Neurol Sci. (2019) 40:2447–57. doi: 10.1007/s10072-019-04015-x 31367861

