# Correction: Deep learning based optic nerve sheath diameter characterization and structure quantification on transorbital ultrasound images

**DOI:** 10.3389/fmed.2026.1790647

**Published:** 2026-02-06

**Authors:** Miao Yang, Cong Liu, Pingyang Zou, Wu Wang

**Affiliations:** 1 Department of Anesthesiology, Guizhou Provincial People's Hospital, Guiyang, China; 2 School of Optical-Electrical and Computer Engineering, University of Shanghai for Science and Technology, Shanghai, China; 3 Electrical Engineering College, Guizhou University, Guiyang, China

**Keywords:** deep learning, optic nerve sheath diameter characterization, optic nerve segmentation, artificial intelligence, medical imaging

The title of this article was erroneously given as: Deep Learning-based Optic Nerve Diameter Sheath Characterization and Structure Quantification on Transorbital Ultrasound Images. The correct title of the article is Deep Learning based Optic Nerve Sheath Diameter Characterization and Structure Quantification on Transorbital Ultrasound Images.

The original version of this article has been updated.

